# Comparative Analysis of Social Networks in Institutionalized Older Adults versus Aging-in-Place Scenarios

**DOI:** 10.3390/geriatrics9010018

**Published:** 2024-02-01

**Authors:** Constantin Ciucurel, Mariana Ionela Tudor, Manuela Mihaela Ciucurel, Ioan-Cosmin Boca, Elena Ioana Iconaru

**Affiliations:** 1 Department of Medical Assistance and Physical Therapy, University Center of Pitesti, National University for Science and Technology Politehnica Bucuresti, 110040 Pitesti, Romania; constantin.ciucurel@upb.ro (C.C.); mariana.tudor0610@upb.ro (M.I.T.); elena_ioana.iconaru@upb.ro (E.I.I.); 2 Department of Psychology, Communication Sciences and Social Assistance, University Center of Pitesti, National University for Science and Technology Politehnica Bucuresti, 110040 Pitesti, Romania; manuela.ciucurel@upb.ro; 3 Department of Physical Education, Sport and Physical Therapy, University of Oradea, 410087 Oradea, Romania

**Keywords:** social connections, institutional care, aging independently, comparative social analysis, social engagement and isolation, social support

## Abstract

(1) Background: This research aims to compare social networks among institutionalized and aging-in-place (AIP) older adults through the validation of a new questionnaire. (2) Methods: The cross-sectional study included 100 older adults (mean age: 73.53 ± 5.49 years; age range: 65–85 years), with 48 institutionalized subjects and 52 AIP subjects. We developed, validated, and administered a new questionnaire, the Social Network Assessment for Older People Questionnaire (SNAOPQ), to assess older adults’ social networks using descriptive and inferential statistical methods. (3) Results: The SNAOPQ demonstrated excellent internal consistency (Cronbach’s alpha of 0.91 and McDonald’s omega of 0.91). Statistical analysis revealed significant associations between variables, highlighting differences in social networks between institutionalized and AIP individuals (*p* ≤ 0.001). Sociodemographic factors like age, education, living arrangement status, and number of descendants significantly influenced SNAOPQ scores (*p* ≤ 0.001). Age and residence type notably impacted participants’ scores, indicating reduced social network size with age. Tertiary education and living in a couple were associated with more extensive social networks, while a higher number of descendants correlated with social network expansion. (4) Conclusions: Our study highlights significant differences in social networks among older adults based on residence type, emphasizing the impact of sociodemographic factors such as age, education, living arrangement, and the number of descendants.

## 1. Introduction

The increasing aging population in Europe has profound implications for health care, social security, and the economy. Effective strategies must be implemented to address these challenges, accommodating shifting demographics and ensuring the well-being and integration of older individuals into society. Europe’s population is rapidly aging, reaching 21.1% aged 65+ in 2022 [1]. The growing number of older adults, especially those 80+, poses economic challenges, with the old-age dependency ratio projected to double by 2100 [1]. Moreover, aging is accompanied by a higher prevalence of chronic diseases, leading to complex health issues that demand careful management [2]. These factors highlight the challenges associated with addressing the health-related aspects of aging.

In Romania, a rapid aging trend underscores the urgent need for comprehensive social and economic policies to bridge generational gaps and maintain societal stability [3]. The 65-and-above age group is projected to increase from 18.6% in 2022 to 27.5% in 2050, with noticeable urbanization from 45.5% rural in 2022 to 66.7% urban in 2050 [4]. Although most in this age group report good health, disparities based on income and living environment persist. Approximately 46% of older people grapple with chronic diseases, while 31% encounter constraints in daily activities [5]. This emphasizes the immediate necessity of tailored interventions addressing cultural nuances and allocating resources efficiently to meet the changing needs of the aging population, with substantial implications for health care, social services, and economic structures.

The demographic data presented herein form the basis for our understanding of the importance of social support networks among the growing population of older adults. Social networks in gerontology, which encompass intricate relationships with family, friends, and the community, are essential for older adults, as they significantly influence their quality of life [6]. These connections provide emotional, instrumental, and informational support, enhancing well-being, reducing isolation, and enabling independence through collaboration with formal services, as a premise in creating age-friendly environments [7]. Additionally, the promotion of intergenerational collaboration and age-friendly policies aligns with the function of older people social networks. Integrating these efforts with innovations in elder care establishes a comprehensive approach, ensuring sustainable and supportive environments for aging individuals.

The evolving social support networks of older adults, influenced by life events and social transitions, significantly impact their health and are essential in community health care [8]. The role of perceived social support in improving the mental health of older individuals has been widely discussed, focusing on its connection to social interaction and psychological well-being [9]. Social support was found to partially mediate the connection between social networks and self-rated health in older adults, highlighting that the strengthening of relationships contributes to their overall mental and physical well-being [10].

Building upon the concept of social networks among older adults, the next step is to analyze the various social aging options, considering the opposite approaches of aging in place (AIP) and institutionalization. AIP refers to older adults living independently in their own homes, preserving familiar environments and social connections [11]. Conversely, institutionalization entails moving older adults to facilities like nursing homes, offering structured care but possibly disrupting familiar surroundings and connections. The decision between these options relies on individual needs, preferences, and the support systems available [12].

Cultural factors determine the preference for AIP, as they are correlated with attitudes and behaviors regarding community and home environments, with older individuals striving to preserve their cultural identity and traditions [13]. Beyond cultural factors and closely related to them, physical environmental factors must also be taken into account. The idea of creating age-friendly communities to enhance residents’ physical well-being is closely linked to the significant impact of social connectedness and neighborhood satisfaction on older adults’ desire to age in place [14]. Therefore, strategies for community-level planning and interventions in promoting healthy aging among older adults become a priority.

Understanding social networks is necessary to tackle loneliness and promote community cohesion [15]. Direct or even virtual interactions among older adults are key to building a healthy global community. For older adults, interpersonal connections, especially regular interactions with family and friends, provide emotional support, combat loneliness, and maintain cognitive function [16,17].

Often, social connections that involve face-to-face interactions are limited in their possibilities. Fortunately, contemporary society has transitioned toward virtual spaces, marking a novel phenomenon confronted by humanity. In the digital age, online platforms enable social interactions for older individuals, providing virtual socialization and support. This form of socialization presents several challenges, such as digital literacy and social exclusion, which are aspects that need to be considered when analyzing phenomena related to social inclusion and social deprivation. Studies indicate that digital social participation decreases with age, particularly after 85. Females, individuals with higher education, and those living together are more active participants in digital platforms [18].

The importance of virtual networks has significantly increased in recent years due to the COVID-19 pandemic, which has led to a restriction of direct social contacts [19]. In general, older people’s challenging living conditions highlight issues like financial insecurity, limited health care, declining health, and social problems. COVID-19 worsened these challenges, especially for older individuals, making specific policies essential during and after the pandemic. The relevance of the proposed research becomes even clearer in the post-pandemic context, where older adults continue to live and adapt to the intersection between traditional customs and the new horizons of technological development.

Lastly, an aspect that needs to be discussed concerns the assessment method of older adults’ social networks. The literature is abundant on this topic [20]. Various tools have been suggested to measure both the size and quality of social networks among older adults, but their suitability is contingent upon specific cultural contexts [21,22]. The evaluation of older adults’ social networks requires a detailed analysis, considering both qualitative and quantitative dimensions to guide effective interventions and support their well-being [23]. Given the existing controversies in our current comprehension of social networks in gerontology [24], most instruments designed for such evaluations diverge in terms of the number of distinct domains they assess. This divergence is observed in both quantitative and qualitative constructs, as well as in the types of alters considered and the specific study contexts [23]. Measuring social networks can involve focusing on an individual (node) or examining the complete networks of entire groups of nodes [25]. The choice between individual-level and network-level measures is contingent upon the research goals and the desired depth of understanding regarding the social dynamics within the studied group [26].

Taking into consideration the diverse landscape in the study of social networks in older adults, there is a need to develop a standardized measure to explore various network types [27]. A gap has been identified in the literature context, specifically concerning the development of studies on social network typology with large samples and a versatile and multidimensional data collection instrument [28].

The aim of this study is to investigate and compare the social interactions of institutionalized and AIP older adults, focusing on identifying differences in the structures and quality of their social networks. This investigation provides valuable insights into diverse aging experiences and contexts. The assessment of social networks involves designing, validating, and administering a targeted questionnaire, reflecting the size and quality of social networks in both categories of older people. The results of this study have the potential to support significant improvements in the quality of life for older adults by addressing deficiencies in social networks, promoting healthy aging, supporting autonomy, and tailoring services to specific social needs in contemporary communities.

For this study, we formulated the following research hypotheses:

1. The newly designed questionnaire will exhibit consistent internal reliability in assessing the social network structures of older adults.
2. Administering the validated questionnaire will reveal nuanced differences in the size and quality of social networks among institutionalized and AIP older adults, offering insights supporting the development of solutions to enhance support services for this targeted group.

The rationale of the study stems from the intention to improve our comprehension through a detailed exploration of social interactions among institutionalized and AIP older adults, providing insights into the structure and quality of their social networks. The utilization of an advanced assessment tool, with its multidimensional approach, adds novelty to the study, facilitating a nuanced analysis of older adults’ social dynamics. The envisioned outcomes will not only add depth to academic discourse but also inform practical solutions to enhance the support services tailored to the unique needs of this demographic group.

## 2. Materials and Methods

### 2.1. Participants and Study Design

This study was based on a cross-sectional research design, involving a total of 100 participants aged 65 and above (mean age: 73.53 ± 5.49 years; age range: 65–85 years), with a female-to-male sex ratio of 1.5:1. The sample comprised 48 older adults residing in institutional care facilities (Group 1, institutionalized) and 52 individuals living independently in their own homes (Group 2, AIP).

In constructing the study sample, the inclusion criteria were as follows: participants aged 65 years and above, permanent residency in the last year (either in a residential facility or at home in urban areas), and willingness to participate in the study. Additionally, a minimum literacy level was considered to ensure participants had the ability to comprehend and respond to the questionnaire items coherently and appropriately. To ensure the accuracy and reliability of the study data, exclusion criteria were applied, such as individuals with severe cognitive impairments limiting communication, a history of acute medical conditions requiring immediate attention, a history of psychiatric disorders, older adults receiving medication or treatments that could affect their social interactions, and a history of substance abuse or addiction.

The recruitment and selection of participants took place over a period of two months during the summer season. We chose this time frame because social interaction is influenced by the season, with a higher tendency for social isolation among older adults during cold periods [29]. Participants were recruited through collaboration with local senior centers, nursing homes, community organizations, and families caring for older adults. This study was conducted following ethical guidelines, ensuring participant confidentiality, anonymity, and informed consent.

### 2.2. Data Acquisition

As an initial step, the research design included the gathering of categorical sociodemographic data about the participants, covering age, gender, residence status (institutionalized or AIP), educational background (primary, secondary, or tertiary education), living arrangement status (living alone or living in a couple), and number of descendants (none, one, two, or more than two), along with relevant medical information concerning current diagnoses and personal medical history. Regarding living arrangement status, we differentiate individuals into two groups: those living in a couple and those living alone. The group of individuals living in a couple includes individuals who are either married; in a long-term, committed relationship; or cohabiting, while the group of individuals living alone comprises individuals who are unmarried, widowed, or formally married but living without a partner. Our decision to consider individuals living as a couple, regardless of marital status, aligns with research showing that cohabiting and married couples share similar relationship dynamics [30]. To gather these data, a brief anamnestic interview and a review of the participants’ medical records were conducted, determining their eligibility for inclusion in the study sample.

In the second phase, following a technical briefing, a questionnaire assessing the individuals’ social network was administered (Social Network Assessment for Older People Questionnaire, SNAOPQ). The questionnaire was either presented directly in written form, if feasible, or administered by an interviewer who posed and explained the questions and response options to the participant. Then, the interviewer marked the selected responses based on the participant’s answers.

SNAOPQ was developed through several stages. In the design phase, we formulated a comprehensive questionnaire specifically tailored for evaluating the size and quality of social networks among older adults. This process included an in-depth review of relevant bibliographic references and expert input to incorporate validated and widely recognized dimensions of social networks. To enhance the questionnaire’s precision and relevance, pilot testing was conducted with a small group of participants. The feedback obtained during this phase was invaluable in identifying areas that required refinement and modification. These essential revisions were made to address any ambiguities, improve clarity, and ensure that the questionnaire aligned with the intended objectives of assessing social networks among older adults. The instrument’s reliability was assessed using widely recognized statistical methods involving both Cronbach’s alpha coefficient and McDonald’s omega coefficient. Following the iterative refinement process, the finalized version of the questionnaire, SNAOPQ, was administered to the study participants.

The final version of SNAOPQ is structured to encompass five relevant dimensions of social interactions:

1. Family and relationships with relatives: This domain pertains to the frequency and intimacy of communication with close family members, the receipt of emotional or material support, and overall satisfaction levels with the support received. It involves a detailed exploration of interpersonal dynamics within the familial context, assessing the depth of connections and evaluating the satisfaction derived from familial relationships and support structures.
2. Friends and acquaintances: In this section, participants provide insights into the number and frequency of interactions with close friends, as well as the quantity and intensity of communication with acquaintances, capturing the broader spectrum of social connections beyond familial relationships.
3. Social activities and community involvement: In this section, participants share insights into their social engagement and community participation. This encompasses the frequency of involvement in social events, participation in discussion groups or clubs for older people, engagement in community activities or volunteer groups, and self-perceived integration within the local community.
4. Social and emotional support: This domain examines individuals’ perceptions of their social connections. It gauges the presence of confidants during challenges, the frequency of loneliness, openness in discussing emotions, and perceived support in difficult situations, offering insights into participants’ emotional and social dynamics.
5. Technology and online communication: Through this section, we aimed to delve into individuals’ adept use of digital tools for interpersonal connections. By examining the frequency of video calls, text messaging, and online social network use to connect with friends, family, or caregivers, as well as gauging their comfort levels with technology for staying connected, this domain aims to provide insights into participants’ engagement with digital communication methods and their overall comfort in utilizing technology for social interactions.

It should be noted that our questionnaire, which was designed for the assessment of the social networks of older adults, introduces a unique perspective by incorporating dimensions of virtual (digital) communication. The distinctive structure of our questionnaire presents challenges in identifying a directly comparable tool, and for this reason, we were unable to undertake a comparison process with subsequent analysis.

The questionnaire utilizes a Likert scale to rate responses, with possible responses ranging from 1 to 5. Each question is assigned a numerical value based on the participant’s response, ranging from 1 to 5, increasing in intensity. Four questions were allocated for each of the five mentioned dimensions of the questionnaire. The scores are then summed within each section, providing a maximum possible score of 20 for each category and a maximum total score of 100. Higher scores indicate more frequent social interactions, stronger emotional support, and greater comfort with technology. This structured approach enables a detailed analysis of each participant’s social network characteristics and, overall, at the sample level, allows for a nuanced analysis, enabling researchers to identify patterns, strengths, and areas for improvement in older adults’ social networks.

### 2.3. Statistical Processing of Data

During the data collection phase, responses were obtained from both institutionalized and AIP older adults. Subsequently, a systematic analysis was conducted on the gathered data to identify patterns and disparities in social interactions between the two groups. The statistical procedures employed a range of techniques. Descriptive methods involved computing means, standard deviations, and frequencies, as well as assessing data normality using the Shapiro–Wilk test. Inferential methods involved evaluating the internal consistency of the questionnaire using Cronbach’s alpha coefficient and McDonald’s omega coefficient [31], as well as the chi-square test of association to establish the statistical significance of subject distribution across the groups concerning gender, educational level, living arrangement status, and number of descendants. To further analyze the data, parametric methods such as Pearson’s linear correlation were employed. Finally, a one-way ANCOVA with Bonferroni’s post hoc test and a two-way ANOVA with Tukey’s post hoc test were utilized.

## 3. Results and Discussion

The presentation of the results begins with the general characterization of the two subgroups of participants (Group 1 and Group 2) concerning gender distribution, educational status, living arrangement status, and the number of descendants (Table 1). The mean age in Group 1 was 73.21 ± 5.41 years, while that in Group 2 was 73.83 ± 5.60 years. It is noteworthy that all participants are retirees and no longer engage in paid professional activities.

We applied the chi-square test to the variables listed in Table 1 to assess the distribution of subjects in the two groups based on gender, educational level, living arrangement status, and number of descendants. The results did not yield enough statistical evidence to reject the null hypothesis regarding gender, educational status, and the number of descendants, suggesting a lack of statistical support for a significant difference between Group 1 and Group 2 concerning these variables. In this specific study and sample, the choice between institutionalized care and AIP is not associated with significant differences in the sociodemographic characteristics of older adults, but caution is advised in generalizing these findings to broader populations without further research and analysis.

Instead, living arrangement status is significantly associated with group affiliation (χ² = 4.18, *p* ≤ 0.034), confirming an anticipated outcome. The institutionalized setting typically accommodates individuals living alone, whereas those AIP often have a higher likelihood of residing as couples. In this regard, single older adults who do not live in a couple face higher risks of long-term care admission. On the other hand, living as a couple may provide protection against institutionalization, enabling individuals to age in place at home [32]. To mitigate the higher risk of long-term care admission for single older adults, the promotion and development of home-based support programs and services are recommended. Such initiatives can assist in managing daily needs and fostering social connections, enhancing quality of life and promoting the ability to age in place at home.

In the process of assessing the questionnaire’s internal consistency, we initially computed Cronbach’s alpha coefficient, resulting in a value of 0.91. This value indicates excellent internal consistency among the questionnaire items [33] and highlights the robustness and reliability of the instrument. We then calculated McDonald’s omega coefficient; the resulting value of 0.91 also suggests a high level of internal consistency [31].

Another aspect of the descriptive statistical analysis involved calculating the measures of central tendency for the SNAOPQ scores (mean and standard deviation) for study participants in both groups, categorized into subgroups based on the considered sociodemographic factors (Table 2).

In the subsequent stage, we calculated the Pearson correlation coefficient between age and SNAOPQ scores within the merged groups, yielding a statistically significant value of −0.44 (*p* ≤ 0.001). This result indicates a moderate relationship between age and SNAOPQ scores, implying that as age increased, the SNAOPQ scores tended to decrease within the entire sample. Our findings are consistent with prior research indicating a decline in social network size with age [16]. This decrease may be attributed to factors such as the loss of close relationships, limited social interactions, and heightened feelings of loneliness, as well as changes in health status, mobility limitations, and retirement transitions [15]. Given this result, we recommend the implementation of social programs and interventions to support the establishment and upkeep of social networks for older adults. Such initiatives can alleviate the negative effects linked to the decline in social network size with age, promoting well-being and reducing the risk of social isolation and loneliness.

The subsequent analysis involved comparing the mean SNAOPQ scores between institutionalized participants (Group 1) and those AIP (Group 2), taking into account the considered sociodemographic variables. To achieve this, we initially determined the distribution pattern of SNAOPQ scores in the two groups and confirmed data normality using the right-tailed Shapiro-Wilk test. Next, we conducted a one-way ANCOVA to explore the relationship between SNAOPQ scores, participants’ age, and their group affiliation (institutionalized or AIP). Utilizing age as a covariate and considering group membership as a fixed factor, our aim was to investigate the combined impact of age and residency mode on the SNAOPQ scores.

**Figure 1:**
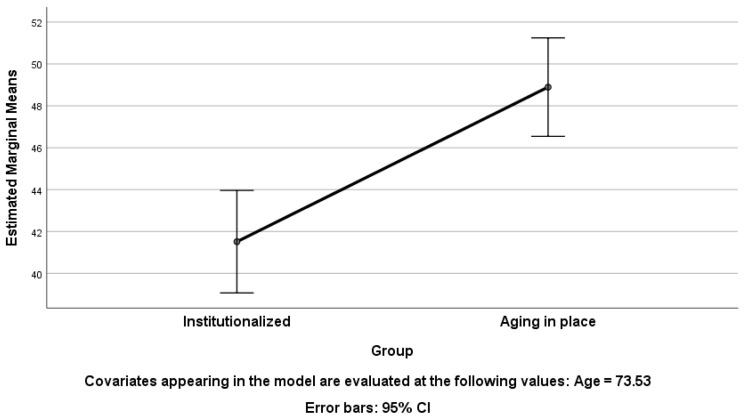
Estimated marginal means of SNAOPQ scores (one-way ANCOVA; dependent variable, SNAOPQ score; fixed factor, residency mode; covariate, age).

Through ANCOVA, we identified significant influences of both age and group membership on participants’ SNAOPQ scores, highlighting notable differences in their social networks (Table 3 and Figure 1). The effect sizes, represented by large partial eta squared values (>0.14) [34], emphasize the substantial impact of these factors. Specifically, age explains 24% of the variation in SNAOPQ scores, while group membership (institutionalized vs. AIP) accounts for 16% of the score variance. Moreover, the observed power values reinforce the robustness of our findings (1 and 0.99 for Age and Group, respectively). It is worth noting that we conducted our analysis with participants aged between 65 and 85 years, and the results should be reported for this specific age group. This ensures the relevance and specificity of our findings within this particular segment of the population.

Within the ANCOVA analysis, we conducted a Bonferroni post hoc test (Table 4). The results indicate significant differences between the institutionalized group and those AIP concerning the mean scores on SNAOPQ (*p* ≤ 0.001). The institutionalized group obtained a significantly lower average score than the AIP group.

The relationship between the size of older adults’ social networks and the type of residence, with age as a covariate factor, can be interpreted through the lens of socioemotional selectivity theory [35]. Therefore, older adults tend to maintain smaller social networks, as they prioritize closer relationships [36]. This perspective aligns with our findings and is particularly relevant to the observed narrowing of social networks with age among older adults. Moreover, some authors suggest a correlation between the size and satisfaction of social networks, both influencing the overall quality of life among older adults [21]. The shift from social network size to its quality underscores the significance of these elements in determining an individual’s quality of life across diverse existential contexts and ontogenetic stages. Considering these factors, it highlights the importance of actively promoting the improvement and quality of social networks for older adults, emphasizing not only their size but also fostering deeper connections to enhance overall life satisfaction.

**Figure 2:**
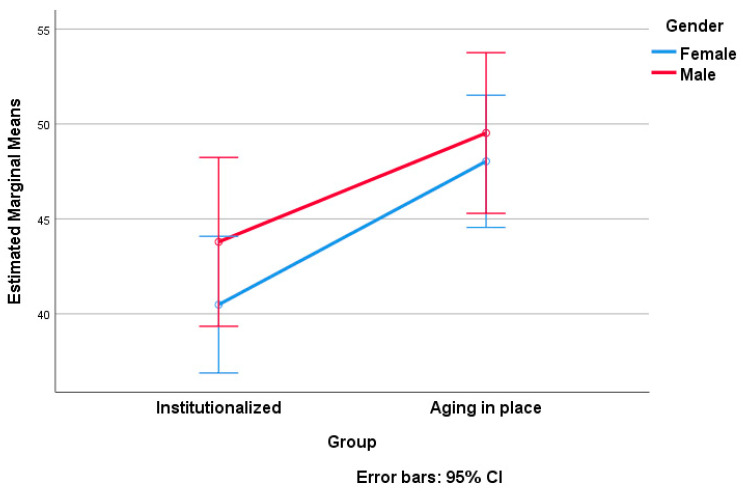
Estimated marginal means of SNAOPQ scores (two-way ANOVA; dependent variable, SNAOPQ score; factor 1, group; factor 2, gender).

**Figure 3:**
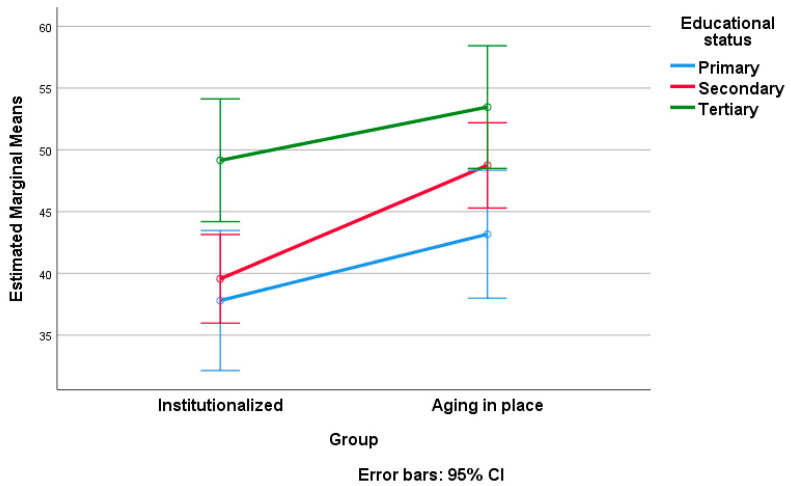
Estimated marginal means of SNAOPQ scores (two-way ANOVA; dependent variable, SNAOPQ score; factor 1, group; factor 2, educational status).

**Figure 4:**
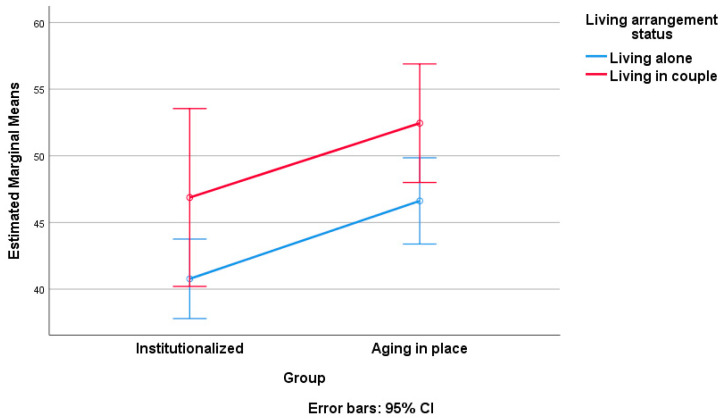
Estimated marginal means of SNAOPQ scores (two-way ANOVA; dependent variable, SNAOPQ score; factor 1, group; factor 2, living arrangement status).

**Figure 5:**
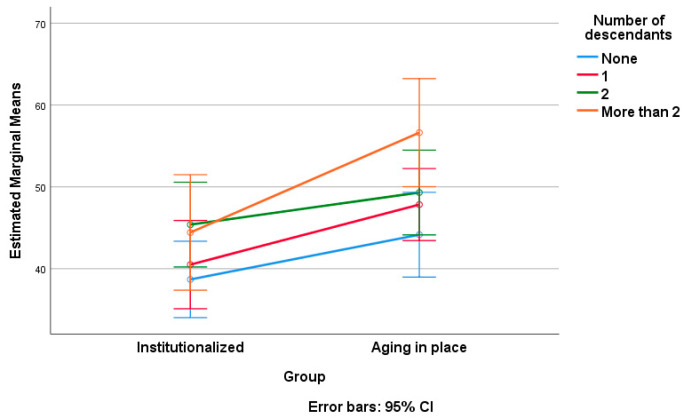
Estimated marginal means of SNAOPQ scores (two-way ANOVA; dependent variable, SNAOPQ score; factor 1, group; factor 2, number of descendants).

At the end of our data processing, we applied a two-way ANOVA, considering the score on SNAOPQ as the dependent variable and group affiliation (institutionalized versus AIP) as factor 1. As factor 2, we examined the influence of sociodemographic variables, including gender, educational status, living arrangement status, and number of descendants, one at a time (Table 5, Table 6, Table 7, Table 8, Table 9 and Table 10). Table 5, Table 6, Table 8 and Table 9 present the results of tests of between-subject effects for each of the considered sociodemographic factors. Note that post hoc Tukey tests were conducted only for factors with more than two levels of variation (educational status and number of descendants; see Table 7 and Table 10, respectively). Figure 2, Figure 3, Figure 4 and Figure 5 visually depict the estimated marginal means of SNAOPQ scores for the two-way ANOVA analyses.

The first notable aspect revealed by the two-way ANOVA analysis was that the social networks of older adults, quantified in terms of SNAOPQ scores, are not significantly influenced by gender or the interaction between gender and residence type. Instead, only the type of residence was found to be statistically significant in the reported analysis, with the partial eta squared indicating a moderate effect size and explaining 10% of the variation in SNAOPQ scores. The observed power for the same factor was 0.91, demonstrating a high level of statistical power. This finding diverges from existing literature suggesting gender differences in social network patterns, at least at the level of utilizing digital social platforms [37,38]. This discrepancy can be attributed to the unique dynamics of social interactions in older people, where cultural, educational, life experience, or other factors may hold more significance than simple gender categorization. On the other hand, it is known that gender-specific motivation patterns, influenced by traditional gender roles [39], affect psychological well-being indicators and social engagement.

Consequently, according to our findings, the gender–social network connection is not as clearly defined between institutionalized and AIP individuals, mitigating the influence of traditional gender stereotypes. As a practical implication, this result suggests directions of action, such as reconsidering the design of targeted interventions to address the nuanced influence of residence type on the social networks of older adults, diverging from conventional gender-based expectations.

Considering the educational factor, we identified significant differences among subgroups with different levels of education (between those with primary education and those with tertiary education, as well as between those with secondary education and those with tertiary education) in both types of residency. Therefore, SNAOPQ scores tend to increase with individuals’ level of education, without significantly interfering with the type of residence. In the given context, the partial eta squared values demonstrate a significant impact, with residence type explaining 10% of the variation in SNAOPQ scores, while educational status accounts for 17% of the variation. For this analysis, the observed power was 0.90 for residence type and 0.97 for educational status. These values indicate a high level of statistical power, suggesting the reliability and robustness of the obtained results for both variables.

The distinctive impact of higher education in shaping social connections among older adults is underscored by its ability to cultivate expansive social networks, as evidenced in both institutionalized and AIP settings. The relationship between education level and the social network was clearly delineated concerning the concept of successful aging [40]. Moreover, it appears that higher education is associated with better health outcomes, as indicated by recent research [41]. Hence, we can anticipate that a well-developed social network provides optimal conditions for aging, with a maximum potential for health, particularly when the individual has invested in higher education. In other words, this analysis suggests that, regarding the educational factor, there is a need to develop personalized strategies to support the social networks of older adults based on their education levels. Therefore, we recommend that interventions focus on promoting education and cultivating social networks in an adapted manner, recognizing the significant contribution of higher education the expansion of such connections.

The next noteworthy result is related to the impact of residence type and living arrangement status (independent variables) on the SNAOPQ scores (dependent variable). As previously mentioned, both independent variables significantly affect the dependent variable, whereas their interaction does not. The partial eta squared values indicate a moderate but statistically significant influence of both factors (residence type accounting for 6% and living arrangement status accounting for 7%) on the variation in SNAOPQ scores. Moreover, the moderate observed power values (0.69 for residence type and 0.73 for living arrangement status) emphasize the robustness and reliability of these results. The conclusion is that living in a couple fosters a larger social network, regardless of the type of residence. This phenomenon can be attributed to the mutual support, companionship, and increased social opportunities provided by having a partner [42], leading to a more extensive social network among older adults. Furthermore, the presence of a life partner tends to stimulate individuals to be more engaged in various activities [43] and probably contributes to the expansion of older adults’ social networks, whether they are institutionalized or not.

In practical terms, it is relevant to consider the influence of residence type and living arrangement status on the social networks of older adults. Therefore, for couples, the emphasis is on strengthening and maintaining supportive aspects in the relationship, promoting shared activities, and encouraging social engagement as a unit. In the case of individuals not living in a couple, recommendations highlight the importance of building connections within broader social circles, participating in community activities, and exploring avenues for meaningful social interactions to offset potential isolation.

The last discussion focuses on the influences of residence type and the number of descendants on SNAOPQ scores. Once again, each factor has individually statistically significant effects on the SNAOPQ score, but their interaction does not. Thus, the partial eta squared values reveal moderate effects, with residence type accounting for approximately 13% of the variance in SNAOPQ scores, while the number of descendants explains around 11% of the variance. The observed power values for the two factors are 0.95 and 0.79, respectively, which can be interpreted as indicating a high level of statistical power for residence type and a moderate level of statistical power for the number of descendants in predicting SNAOPQ scores. Post hoc analyses, particularly that employing the Tukey test, identified significant variations in SNAOPQ scores among participant subgroups; those without descendants differed significantly from those with two descendants, as well as from those with more than two descendants.

Summarizing the above results, having multiple descendants appears to be necessary to observe a significant increase in the size of the social network among older adults, whether they are institutionalized or not. This suggests that a larger number of descendants may create more opportunities for social interactions, thereby contributing to a more extensive social network, regardless of the type of residence. To explain these findings, we commence with the well-established observation that in the absence of descendants, older individuals are predisposed to solitary living, resulting in diminished social engagement and adverse consequences for their health and quality of life [44]. Furthermore, the presence of a family and a social network built on familial relationships with descendants was recognized as a mitigating factor in the gradual advancement of aging [45].

It is evident that older adults without descendants constitute a socially disadvantaged category, with some authors considering them a vulnerable group towards which specific social support policies should be directed [46]. Building upon the aforementioned findings, it is imperative to sustain interventions that prioritize initiatives fostering family engagement and support networks to enhance social connections among older adults. For those without descendants, targeted programs promoting community involvement, peer support, and organized activities can effectively alleviate the risk of social isolation and its associated negative consequences.

Overall, our study reveals disparities between institutionalized older adults and those AIP, with the former having a more limited social network, influenced by the investigated sociodemographic factors but with certain specificities related to each considered variable. These findings underscore the practical significance of living arrangements in shaping the social networks of older individuals in specific contexts. As a novelty, we incorporated the virtual component of communication within social networks as an element investigated through SNAOPQ, assessing online interactions and connections. This approach provided valuable insights into how digital communication technologies and social media platforms influence the social interactions and networks of older adults.

This study has the potential to significantly impact the quality of care for older adults, regardless of their residence type, by informing personalized caregiving strategies, addressing social network deficiencies, and promoting healthy aging. Additionally, it may influence aging-related policy development, guiding the strategic allocation of resources to support families and caregivers and enhance the autonomy of older adults. Consequently, the results of this study can help to optimize resource allocation, ensuring services are directed where they are most needed. Tailoring services to the specific social needs of older adults, such interventions are designed to enhance autonomy and social participation in various contexts [47]. Understanding social network differences enables targeted resource allocation, enriching older adult care and improving quality of life in contemporary communities [48]. Further research is needed to explore the long-term effects of these interventions and to refine strategies to enhance the social well-being of older adults.

The SNAOPQ stands out from existing measures through its investigation of five comprehensive dimensions. This innovative structure provides a nuanced perspective on the complex nature of older adults’ social dynamics, offering a comprehensive and multidimensional assessment tool. The SNAOPQ’s distinctive approach addresses the limitations of conventional measures and enhances our understanding of the diverse aspects of social interactions among older people.

This study has inherent limitations, especially due to the exclusion of certain socio-demographic variables, such as household size, cohabitation with children, rental status, and other factors that could potentially influence the social networks of older adults. Additionally, the study did not consider geographical variations or cultural differences, which might affect social interactions and support systems differently across regions and communities [49]. Furthermore, the research focused on a specific age group (65 to 85 years old), limiting the generalizability of the findings to younger or older age cohorts. The study also relied on self-reported data, which could introduce response bias and affect the accuracy of the results. Our questionnaire was unable to undergo validation through comparison with another instrument due to its unique structure and the specific dimensions investigated. Other limitations that can be mentioned include the absence of in-depth analysis regarding online interactions; the cross-sectional nature of the study, limiting causal relationships; and the lack of exploration of the impacts of health conditions and disabilities on older adults’ social networks. Additionally, variations resulting from the diverse administration methods of the SNAOPQ (directly in written form or through an interviewer) were not specifically analyzed. These limitations highlight the need for further research to provide a comprehensive understanding of the factors influencing social networks among older adults.

## 4. Conclusions

Our study examined social network differences among older adults based on residence type, comparing institutionalized individuals with those AIP while considering various sociodemographic variables. In the first stage, we validated and demonstrated the excellent internal consistency of the SNAOPQ instrument (Cronbach’s alpha of 0.91 and McDonald’s omega of 0.91). Subsequently, based on the questionnaire administration results, statistical analysis revealed significant associations between the examined variables. First, significant differences in social networks were highlighted between institutionalized subjects and those AIP in terms of SNAOPQ scores (*p* ≤ 0.001). Regardless of the residence type, sociodemographic factors such as age, educational level, living arrangement status, and number of descendants exerted statistically significant influences on SNAOPQ scores (*p* ≤ 0.001). We identified that both age and type of residence significantly influenced participants’ SNAOPQ scores, indicating a decrease in social network size with advancing age. Additionally, older adults with tertiary education exhibited the most extensive social networks, emphasizing the role of education in shaping social interactions. Living in a couple was also associated with richer social networks, while the number of descendants influenced social network size, suggesting an expansion tendency with a higher number of offspring.

These findings underscore the importance of social support and partnership in the lives of older adults, serving as early foundations in the aging process, both at home and in institutional settings. These results offer significant guidance for the development of intervention strategies, emphasizing the necessity of supporting access to education and promoting healthy relationships to enhance the social and emotional well-being of older people. Our conclusions can form the basis for policies and programs aimed at supporting older adult communities, thereby facilitating social interactions and promoting active and satisfying aging.

Overall, the study’s novelty lies in introducing the new SNAOPQ assessment tool, facilitating a detailed examination of social interactions among older adults in different existential conditions. In terms of practical implications, our research provides valuable insights for the development and customization of support services to enhance the well-being of older adults, informed by a comprehensive understanding of their social dynamics through the use of the SNAOPQ.

## Figures and Tables

**Table 1:** Summary of the characteristics of study participants in Group 1 (institutionalized, *n* = 48) and Group 2 (AIP, *n* = 52).

Variable	Categories	Group 1		Group 2	
		Frequency	Percent (%)	Frequency	Percent (%)
Gender	Male	19	39.6%	21	40.4%
	Female	29	60.4%	31	59.6%
Educational status	Primary	10	20.8.1%	12	23.1%
	Secondary	25	52.1%	27	51.9%
	Tertiary	13	27.1%	13	25.0%
Living arrangement status	Living in a couple	8	16.7%	18	34.6%
	Living alone	40	83.3%	34	65.4%
Number of descendants	None	16	33.3%	13	25%
	1	12	25.0%	18	34.6%
	2	13	27.1%	13	25.0%
	More than 2	7	14.6%	8	15.4%

Note—*n*: group size; AIP: aging in place.

**Table 2:** Summary of the SNAOPQ scores for study participants in Group 1 (institutionalized, *n* = 48) and Group 2 (AIP, *n* = 52) depending on sociodemographic factor groupings.

Variable	Categories	Group 1		Group 2	
		Mean	SD	Mean	SD
Gender	Male	40.48	9.27	48.03	10.65
	Female	43.79	9.07	49.52	9.662
Educational status	Primary	37.80	9.11	43.17	10.50
	Secondary	39.56	7.42	48.74	9.01
	Tertiary	49.15	8.92	53.46	10.47
Living arrangement status	Living in a couple	40.78	8.64	46.62	10.35
	Living alone	46.88	11.03	52.44	8.95
Number of descendants	None	38.69	9.99	44.15	10.08
	1	40.50	9.08	47.83	10.01
	2	45.38	8.96	49.31	7.62
	More than 2	44.43	6.50	56.63	11.2
Total		41.79	9.24	48.63	10.19

Note—SD: standard deviation; *n*: group size; AIP: aging in place.

**Table 3:** The results of tests of within-subject effects (one-way ANCOVA; dependent variable, SNAOPQ score; fixed factor, residency mode; covariate, age).

Source	Type III Sum of Squares	df	Mean Square	F	*p* Value	Partial Eta Squared	Observed Power
Age	2242.51	1	2242.51	30.77	0.001	0.24	1
Group	1355.14	1	1355.14	18.59	0.001	0.16	0.99

Note—SNAOPQ: Social Network Assessment for Older People Questionnaire.

**Table 4:** Pairwise comparisons (one-way ANCOVA).

Group I	Group J	Mean Differences (I–J)	Std. Error	*p* Value	95% Confidence Interval for Difference	
					Lower Bound	Upper Bound
Institutionalized	AIP	−7.38	1.71	0.001	−10.78	−3.98

Note—A Bonferroni post hoc test was conducted to compare the main effects.

**Table 5:** The results of tests of within-subject effects (two-way ANOVA; dependent variable, SNAOPQ score; factor 1, group; factor 2, gender).

Source	Type III Sum of Squares	df	Mean Square	F	*p* Value	Partial Eta Squared	Observed Power
Group	1056.70	1	1056.70	11.08	0.001	0.10	0.91
Gender	137.871	1	137.87	1.45	0.232	0.02	0.22
Group*Gender	19.730	1	19.73	0.21	0.650	0.01	0.07

Note—SNAOPQ: Social Network Assessment for Older People Questionnaire.

**Table 6:** The results of tests of within-subject effects (two-way ANOVA; dependent variable, SNAOPQ score; factor 1, group; factor 2, educational status).

Source	Type III Sum of Squares	df	Mean Square	F	*p* Value	Partial Eta Squared	Observed Power
Group	858.28	1	858.28	10.53	0.002	0.10	0.90
Educational status	1512.48	2	756.24	9.28	0.001	0.17	0.97
Group*Educational status	123.25	2	61.63	0.76	0.472	0.02	0.18

Note—SNAOPQ: Social Network Assessment for Older People Questionnaire.

**Table 7:** Multiple comparisons (two-way ANOVA) for educational status.

Educational Status (I)	Educational Status (J)	Mean Differences (I–J)	Std. Error	*p* Value	95% Confidence Interval for Difference	
					Lower Bound	Upper Bound
Primary	Secondary	−3.60	2.296	0.265	−9.07	1.87
	Tertiary	−10.58	2.615	0.000	−16.81	−4.35
Secondary	Primary	3.60	2.296	0.265	−1.87	9.07
	Tertiary	−6.98	2.168	0.005	−12.14	−1.82
Tertiary	Primary	10.58	2.615	0.000	4.35	16.81
	Secondary	6.98	2.168	0.005	1.82	12.14

Note—A Tukey post hoc test was conducted to compare the main effects.

**Table 8:** The results of tests of within-subject effects (two-way ANOVA; dependent variable, SNAOPQ score; factor 1, group; factor 2, living arrangement status).

Source	Type III Sum of Squares	df	Mean Square	F	*p* Value	Partial Eta Squared	Observed Power
Group	554.27	1	554.27	6.14	0.015	0.06	0.69
Living arrangement status	605.40	1	605.40	6.71	0.011	0.07	0.73
Group*Living arrangement status	0.32	1	0.32	0.01	0.953	0.01	0.05

Note—SNAOPQ: Social Network Assessment for Older People Questionnaire.

**Table 9:** The results of tests of within-subject effects (two-way ANOVA; dependent variable, SNAOPQ score; factor 1, group; factor 2, number of descendants).

Source	Type III Sum of Squares	df	Mean Square	F	*p* Value	Partial Eta Squared	Observed Power
Group	1194.72	1	1194.72	13.52	0.000	0.13	0.95
Number of descendants	983.93	3	327.98	3.71	0.014	0.11	0.79
Group*Number of descendants	176.58	3	58.86	0.67	0.575	0.02	0.19

Note—SNAOPQ: Social Network Assessment for Older People Questionnaire.

**Table 10:** Multiple comparisons (two-way ANOVA) for number of descendants.

Educational Status (I)	Educational Status (J)	Mean Differences (I–J)	Std. Error	*p* Value	95% Confidence Interval for Difference	
					Lower Bound	Upper Bound
None	1	−3.76	2.45	0.420	−10.17	2.64
	2	−6.21	2.55	0.076	−12.85	0.44
	More than 2	−9.80	2.99	0.008	−17.62	−1.97
1	None	3.76	2.45	0.420	−2.64	10.17
	2	−2.45	2.52	0.766	−9.04	4.15
	More than 2	−6.03	2.97	0.185	−13.81	1.75
2	None	6.21	2.54	0.076	−0.44	12.85
	1	2.45	2.52	0.766	−4.15	9.04
	More than 2	−3.59	3.05	0.643	−11.56	4.39
More than 2	None	9.80	2.99	0.008	1.97	17.62
	1	6.03	2.97	0.185	−1.75	13.81
	2	3.59	3.05	0.643	−4.39	11.56

Note—A Tukey post hoc test was conducted to compare the main effects.

## Data Availability

The data are available from the corresponding author upon request. All data relevant to the study are included in the article.
